# P-2005. Long-term complications of COVID-19 in pediatric population in Lima, Peru

**DOI:** 10.1093/ofid/ofae631.2162

**Published:** 2025-01-29

**Authors:** Rafaella Navarro, Theresa Ochoa, Gabriel Carrasco, Brian Peña

**Affiliations:** UPCH, Lima, Lima, Peru; Instituto de Medicina Tropical Alexander von Humboldt, Universidad Peruana Cayetano Heredia, Lima, Lima, Peru; UPCH, Lima, Lima, Peru; UPCH, Lima, Lima, Peru

## Abstract

**Background:**

Peru is the eighth country in South America with most COVID-19 cases (134 684 cases per million population) and the first country in the world with most COVID-19 deaths (6 595 deaths per million population). However, is lacking data on the long term complications of COVID-19 in children and adolescents. Peru does not have guidelines for the diagnosis and management of long-term complications of COVID-19. Thus, studies are needed to develop evidence-based management and to propose the necessary infrastructure to provide comprehensive care.

**Figure:**
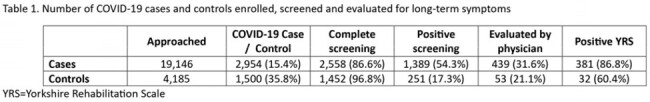

**Methods:**

A multicenter study was carried out in 16 hospitals from Lima to describe the long-term complications of COVID-19 in subjects under 18 years and to compare with subjects without a history of COVID-19 (controls). A history of COVID-19 episode was defined by a positive laboratory test or presence of symptoms with a confirmed household case. The comparison group included subjects with no history of COVID-19 symptoms, no history of COVID-19 epidemiological contact and/or negative laboratory tests. Enrolled patients were screened using a survey from the Long-COVID Clinic of Miami. If the screening was positive the Yorkshire Test was applied during a medical evaluation.

**Figure:**
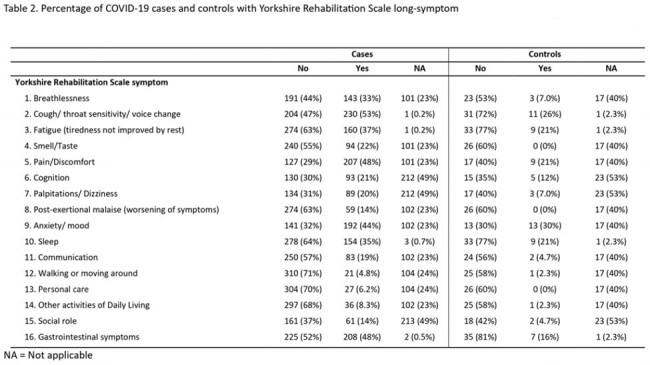

**Results:**

2,954 cases and 1,500 controls were enrolled, from which 2,558 cases and 1,452 controls completed the screening survey. A higher positive screening for long-term symptoms was found in subjects with COVID-19 history (1,389/2,558, 54.3%) in comparison with subjects without COVID-19 history (251/1452, 17.3%). Among subjects with a positive screening, 439 (31.6%) cases and 53 (21.1%) controls completed a medical evaluation; 381 (86.8%) cases had at least one new symptom posterior to COVID-19 that lasted at least 3 months (positive Yorkshire Rehabilitation Scale) and 32 (60.4%) among controls. (Tab 1) The most common symptoms were cough (53% vs 26%), pain (48% vs 21%), gastrointestinal symptoms (48% vs 16%), and anxiety (44% vs 30) among cases and controls, respectively. (Tab 2)

**Conclusion:**

More than half of pediatric patients with COVID-19 have long-term symptoms. Study limitations are: recall bias, not all subjects with a positive screening were evaluated by a physician, and we could be overestimating the frequency of symptoms since we enrolled subjects in hospitals.

**Disclosures:**

Rafaella Navarro, n/a, Pfizer: Grant/Research Support Theresa Ochoa, PhD, Pfizer: Grant/Research Support Gabriel Carrasco, n/a, Pfizer: Grant/Research Support Brian Peña, n/a, Pfizer: Grant/Research Support

